# Assessment of Medical Test Overuse and Its Impact on Pediatric Emergency Department Outcomes in Upper Respiratory Tract Infections in a University Hospital in Lithuania

**DOI:** 10.3390/diagnostics14100970

**Published:** 2024-05-07

**Authors:** Melita Nedzinskaite, Dagna Karakaite, Erika Zubrickyte, Lina Jankauskaite

**Affiliations:** 1 Department of Pediatrics, Lithuanian University of Health Sciences, 44307 Kaunas, Lithuania; lina.jankauskaite@lsmu.lt; 2 Faculty of Medicine, Lithuanian University of Health Sciences, 44307 Kaunas, Lithuania; dagna.karakaite@gmail.com (D.K.); zubrickyte.erika@gmail.com (E.Z.); 3 Department of Pediatrics, Lithuanian University of Health Sciences Kaunas Clinics, 50103 Kaunas, Lithuania

**Keywords:** upper respiratory tract infections, pediatric emergency department, medical overuse, viral infections

## Abstract

Medical overuse poses potential risks to patients and contributes to increasing healthcare costs, pediatric emergency departments (PED) in particular. Often, upper respiratory tract infection (URTI) cases are viral-induced and self-limiting, and they do not require specific investigations or treatment. We conducted a retrospective study from 1 December 2021 to 31 January 2022, thereby aiming to identify the common tests and factors influencing specific diagnostic and treatment decisions for URTI in PED. In total, 307 (74.9%) URTI cases underwent complete blood count (CBC) tests, 312 (76.1%) were subjected to C-reactive protein (CRP) tests, and 110 (26.8%) received urinalysis tests. Patients with a longer duration of fever and a physician‘s suspicion of bacterial infection were more likely to receive CBC, CRP, and/or urinalysis tests (*p* < 0.05). Moreover, 75.1% of the cases were classified as viral URTIs, 9.8% were bacterial URTIs, and 15.1% were unspecified. Notably, 86 (20.1%) children received antibiotics and antibiotic prescription correlated with age, tonsillitis diagnosis, CRP values higher than 30 mg/L, and a CBC of *p* < 0.05. Patients triaged in the second or third categories were three times more likely to be observed for 24 h compared to patients with URTI and the fourth triage category (*p* < 0.05). This study highlights the need for interventions to improve the appropriateness of emergency service utilization, thereby emphasizing the importance of judicious decision making in managing pediatric URTIs.

## 1. Introduction

Medical overuse is characterized by healthcare interventions that provide no overall benefit to patients while being associated with significant costs and potential harm. Despite improvements in diagnostics, as well as diagnostic tools and protocols, pediatric patients are highly affected by medical overuse [1,2]. Unlike adults, children are more vulnerable, exhibit fewer specific symptoms, and experience rapid changes in disease progression [3]. Other factors, such as the reduction of uncertainty or expectation from the patient’s family, are associated with overdiagnosis and overtreatment in pediatric patients [4]. Those factors contribute to unnecessary care, resource utilization, and, in some cases, may even harm the patient [5].

Upper respiratory tract infections (URTIs) constitute a significant portion of non-urgent visits to the pediatric emergency department (PED) [6]. Since most URTIs are viral and self-limiting, advanced testing or specific treatment is generally unnecessary [7]. Patients presenting to PED with URTIs may exhibit a broad spectrum of symptoms that overlap with other diseases. While most viral infections manifest with mild symptoms, the presentation varies among individuals [8,9]. The literature suggests that most physicians may fear missing serious conditions, thus leading to excessive testing and treatment [1]. Harmless intent has been proven to result in harmful outcomes, including a prolonged disease course, more serious issues, and the labeling of chronic diseases [10]. In contrast, presenting to PED too early, or in cases of repeated visits, places URTI patients at risk of undergoing excessive examination, hospitalization, and inappropriate antibiotic use [11].

In this study, we aimed to elucidate the frequency of laboratory tests, such as complete blood count (CBC), C-reactive protein (CRP), urinalysis, chest X-ray, and other diagnostic tests performed in our PED for children diagnosed with URTI. Additionally, we aimed to investigate the factors influencing a physician’s decision to perform diagnostic tests and the factors affecting outcomes (discharge, hospitalization, antibiotic prescription, etc.) for children diagnosed with URTIs at PED.

## 2. Materials and Methods

### 2.1. Study Design and Study Population

A single-center, cross-sectional study was conducted in the pediatric emergency department of the Lithuanian University of Health Sciences Kaunas Clinic’s tertiary hospital. This hospital typically sees approximately 40,000 pediatric visits annually. Data from all the patients diagnosed with upper respiratory tract infections between 1 December 2021 and 31 January 2022 were retrieved from the local electronic healthcare data record system. Newborns, children with chronic diseases, congenital pathologies, psychiatric disorders, and repeated visits were excluded from the study.

### 2.2. Data Collection

Further data were collected, including demographics (age and gender), triage categories, time of arrival from the onset of fever, symptoms, auscultation findings, and physiological parameters, such as respiratory rate (RR), body temperature (t°), heart rate (HR), and blood oxygen saturation (SpO_2_). Data were also collected from conducted laboratory tests, such as complete blood count (CBC), C-reactive protein (CRP), urinalysis, blood, tonsil, cerebrospinal fluid cultures (if any), and the results of rapid strep test (if performed). Instrumental tests, such as chest X-ray, abdominal, and/or chest ultrasound were also documented. Final diagnoses included acute upper respiratory tract viral infection, such as nasopharyngitis or pharyngitis, tonsillitis, and laryngitis. Outcomes were classified as discharge, short stay (up to 24 h), hospitalization, and treatment details, including antibiotics and others.

Additionally, tachycardia and tachypnoea were evaluated in accordance with the European Resuscitation Council Guidelines 2021: Paediatric Life Support [12]. Complete blood count reference values were determined based on Pediatric Hematology edited by Robert Arceci et al. [13]. Patient groups were categorized by age, diagnosis, triage category, and the origin of the infection (bacterial and non-bacterial (i.e., viral and non-identified)). Unspecified etiology infections were excluded from the further analysis of the antibiotic prescription rates. Age groups were defined as <6 months old, 6–11 months old, 1–4 years old, 5–11 years old, and 12–18 years old. The origin of the infection was reassessed based on clinical presentation and investigations (if tests were performed). The triage categories were assigned according to vital signs, primary complaint, pain level, severity of the condition, and medical history upon arrival to PED, all in accordance with the Manchester Triage System [14]. Category number was allocated based on urgency, with the first category indicating the highest level of urgency and the fourth signifying the lowest.

### 2.3. Statistical Analysis

Data analysis was conducted using Microsoft Excel and IBM SPSS Statistics, version 29.0 software (SPSS Inc., Chicago, IL, USA) for Windows. The Shapiro–Wilk test was used to assess for a normal distribution. The non-Gaussian distributed data were described by the median and interquartile range (IQR). The descriptive statistics of the qualitative variables are presented in absolute numbers (*n*) and percentages (%). A chi-square (χ2) test or a Fisher’s exact test were conducted to assess the statistical significance of the observed association. A multivariant logistic regression was performed to calculate the relationship between the odds ratio and the coefficient. Spearman’s correlation coefficient was used to evaluate the statistical strength of the association between variables. A *p*-value of <0.05 was considered significant.

### 2.4. Ethical Consent

Permission to conduct the study was obtained from Kaunas Regional Biomedical Research Ethics Committee (BE-2–27). This study was conducted in accordance with the guidelines detailed in the Declaration of Helsinki and Good Clinical Practice Guidelines.

## 3. Results

In total, 410 children with a median age of 2 (1–4) years were included into this study, where 48.8% (*n* = 200) were female. The majority of children were between 1–4 years of age. Most of the patients were triaged as category 4 (not urgent) (*n* = 354, 86.3%) (Table 1). More than three quarters of the patients arrived ≥24 h after the onset of fever. In the majority of cases, upper viral respiratory infections (such as nasopharyngitis or pharyngitis) were diagnosed (*n* = 309, 75.4%). Bacterial infections were diagnosed in less than 10% (*n* = 40, 9.8%). A total of 382 (93.2%) children were discharged home (Table 1).

### 3.1. Diagnostic Tests

About three quarters of the children received complete blood count (*n* = 307, 74.9%) and C-reactive protein (*n* = 312, 76.1%) tests. A normal CBC was observed in 70.7% (*n* = 217) of the cases. The median CRP was 13.8 (5.0–36.8). Urinalysis tests were performed in 110 (26.8%) cases, with 90.9% showing no changes; others were evaluated as contaminated. Seventeen children received a rapid strep test, and it was positive in two, which accounted for 2.8% of all the tonsillitis cases. Out of the 30 (7.3%) children who received a chest X-ray, 60% (*n* = 18) had pathological findings such as obstruction or crackles on auscultation. No chest X-ray was identified as pathological, except for two cases, which were interpreted as suspected infiltration. However, the final diagnosis was formulated as acute upper respiratory tract infection. Despite the apparent URTI symptoms, an abdominal ultrasound was performed in 19 patients without pathological changes (Table 2).

Patients with suspected bacterial infection were 9.3 times more likely to obtain CBC test (OR 9.31, 95% CI 1.24 to 69.72, *p* = 0.030) and 8.5 times more likely to obtain a CRP test (OR 8.57, 95% CI 1.14 to 64.36, *p* = 0.037). The longer the duration of a fever increased the likelihood of receiving a CBC test by 2.5 (OR 2.56, 95% CI 1.18 to 5.56, *p* = 0.017) and a CRP test by 2.9 (OR 2.90, 95% CI 1.29 to 6.53, *p* = 0.010), as seen in Table 3. A statistically significant result was observed in patients who received a urinalysis test when a bacterial infection was suspected (*p* < 0.05). There was no association with age, gender, triage category, fever, and the incidence of urinalysis tests (Table 3).

CBC and C-reactive protein tests were performed on more than 70% (*n* = 260, 73.0% and *n* = 266, 74.7%, respectively) of the fourth triage category patients, over 80% of the children in the third category, and on all the patients who belonged to the second category (Table 4). Thirty-six percent of children in the third category received urinalysis; nevertheless, most of the children in the fourth category also underwent urinalysis. Almost all patients who received a chest X-ray (*n* = 27, 7.6%) or an abdomen ultrasound (*n* = 16, 4.5%) were categorized as non-urgent cases (fourth and third categories); none of the children in the second category underwent these tests. Only patients in the fourth category were subjected to a urine culture (*n* = 3, 0.8%).

In patients with non-bacterial infection, CBC and CRP tests were prescribed in 72.4% (*n* = 268) and 73.8% (*n* = 273) of cases, respectively, and urinalysis were administered for almost a quarter (*n* = 92, 24.9%) of patients. Rapid strep tests, chest X-rays, and an abdominal ultrasounds were administered to more of the children with non-bacterial infections. More children had their blood drawn for CBC and CRP tests, and urinalysis were conducted when URTI was diagnosed (*p* < 0.05) (Table 4).

### 3.2. Outcomes and Antibiotic Use

Antibacterial therapy was prescribed in 86 cases (20.1%). Notably, this prescription practice was observed in both the emergency department and outpatient treatment settings. A higher incidence of antibiotic prescription was observed in cases with non-bacterial infections compared to the bacterial infection group.

Unspecified etiology infections (*n* = 62, 15.1%) were excluded from the further analysis of antibiotic prescription rates. Following the exclusion of unspecified etiology infections, it was observed that 4.9% (*n* = 17) of viral infections and 11.2% (*n* = 39) of bacterial infections were associated with antibiotic prescription rates. A statistically significant finding demonstrated that a higher proportion of patients were prescribed antibiotics when a bacterial infection was suspected, particularly tonsillitis (Table 5). This tendency was common among patients within the age range of 1 to 5 years, in patients where complete blood count or urinalysis were performed, or when C-reactive protein values exceeded 30 mg/L (*p* < 0.05), as shown in Table 5.

The majority of patients with upper respiratory tract infections were discharged from the pediatric emergency department (Table 1). A much smaller number of patients were observed up to 24 h in pediatric ED or were hospitalized in the pediatrics clinic for further treatment (5.6%, *n* = 23 and 1.2%, *n* = 5, respectively).

The data showed that children triaged in the second or third categories were three times more likely to be observed (OR 3.04, 95% CI 1.17 to 7.92, *p* = 0.023), (Table A1). No associations were found between age, gender, duration of fever, diagnosis, laboratory tests, and 24 h observation rates, as shown in Table A1.

The discharge rates were primarily related to the triage category and the origin of diagnosis. Patients triaged in the fourth category were four times more likely to be discharged (OR 4.89, 95% CI 1.65 to 14.54, *p* = 0.004), and they were also most likely to be discharged if a viral infection was suspected (OR 14.00, 95% CI 2.03 to 96.54, *p* = 0.007) (Table A1). No statistically significant associations were found between age, gender, duration of fever, laboratory tests, and discharge rates (Table A1).

As we expected, the hospitalization rates of the patients with URTIs were low in our study and demonstrated no statistically significant results.

## 4. Discussion

Upper respiratory tract infections (URTIs) represent a primary driver of unnecessary medical visits, thus leading to the overuse of diagnostic tests and inappropriate management decisions, such as hospitalization or antibiotic prescriptions. Our study revealed that the majority of URTI cases were non-urgent, viral, and often subjected to unnecessary testing. Clinician decisions to perform laboratory tests were influenced by the duration of the fever and triage categories in the PED. Common unnecessary diagnostic procedures included CBC, CRP, and urine tests. In addition, radiological testing, such as chest X-ray and abdominal ultrasound, was performed on pediatric patients with URTIs, despite these medical tests typically providing no diagnostic value in such cases as none presented urgent or life-threatening conditions.

Studies have indicated that 20–65% of PED visits are likely to be non-urgent or low acuity cases, thus contributing to emergency department overcrowding, which adversely affects the quality of care and increases healthcare system costs [15,16,17,18,19]. In our study, we observed that most URTI cases were triaged as a category 4, indicating non-urgent visits. While we did not measure overcrowding directly, when considering daily statistics, annual visits, staffing level, and referrals to pediatric emergency department, we believe these cases could potentially contribute to the overcrowding of pediatric emergency departments. Parental concerns contribute to the increasing use of emergency departments, thus making the pediatric population the main users of emergency department facilities [15,20,21,22,23,24]. Addressing this issue necessitates parental education, improved availability of primary care, and healthcare provider training in effective communication to reduce unnecessary PED visits.

Clinical decisions regarding URTI etiology and treatment should typically rely on patient symptoms, but high-income settings often respond to laboratory investigations [25]. Our study found that over two thirds of patients with URTIs underwent CBC and CRP to determine the origin of infection. In our study, it was observed that even 26.7% of laryngitis cases had CBC and CRP tests performed, despite diagnosis traditionally being reliant solely on clinical signs. Similar tendencies were noted in tonsillitis cases within our study, wherein 97% cases underwent CBC and CRP tests, even though it was contrary to recommendations advocating for a preference of rapid tests [26]. Excessive laboratory testing, ranging from 36–69% in various regions [27,28,29], is often attributed to physician experience, cognitive bias, parental concerns, and a perceived risk of a rapid deterioration in a child’s condition [28,30,31].

Furthermore, our study identified urinalysis as the third most common laboratory test among patients with URTIs. Urinary tract infections (UTIs) pose a challenge, as young children with UTIs often present with non-specific symptoms shared with a wide range of diseases [32], including URTIs. In our study, 110 (26.8%) patients had a urine test performed, and none of the test results showed pathological findings. Interestingly, three urine cultures were obtained, and all of them were evaluated as normal or contaminated. Our findings indicated that age, gender, triage category, and duration of fever are not associated with the incidence of urinalysis, thus suggesting that urinalysis are performed against the guidelines and latest recommendations on urinary tract infection diagnostics [33,34,35].

Using a rapid strep test alongside the Centor Criteria for diagnosing group A beta-hemolytic *Streptococcus* pharyngitis can prevent unnecessary antibiotic prescriptions [36], thus proving convenient and acceptable to both patients and clinicians while preventing economic damage [37]. Despite recommendations advocating the avoidance of blood test performance, our study revealed that merely 19.7% of tonsillitis cases underwent a rapid strep test. Additionally, none of the physicians documented the results of the Centor score criteria (data not shown), especially in those 17 cases (4.2%) that underwent a rapid strep test. In contrast to our findings, studies performed in European countries reported high rapid test performance rates in patients diagnosed with pharyngotonsillitis [38,39]. Employing rapid antigen tests for group A beta-hemolytic *Streptococcus* pharyngitis is a cost-effective method; it enables physicians to decide on a treatment plan in one appointment, and it could be used in each PED.

We observed that the overall antibiotic prescription rate was 20.1%, which is comparable to the rates in other European countries [40,41]. Overall, 4.9% of the antibiotic prescriptions were associated with viral diseases, thereby representing unnecessary cases, and these were found to be at a much higher rate than reported in other studies [42]. Inappropriate antibiotic prescriptions do not improve patient outcomes but may increase antimicrobial resistance, thus threatening our ability to treat infectious diseases successfully in the future. Persistently high rates of inappropriate antibiotic prescriptions likely result from multifactorial factors, including patient sociodemographic data, signs and symptoms at presentation, patient expectations, and even individual factors of a physician [41].

There is a need to establish local guidelines that emphasize the judicious use of laboratory investigations to effectively utilize limited healthcare resources in our country. Cautious use of laboratory investigations, radiological imaging, and antimicrobials will help reduce hospital management costs.

Certainly, our study has limitations. First, in data records, some physicians did not define the origin of the infection, thus leaving 15.1% (*n* = 62) of cases unclear with respect to whether a physician suspected bacterial or viral infection. Therefore, the antibiotic prescription rates in the non-bacterial infection group could be identified as too high and impact our conclusion of inappropriate antibiotic use in our setting. Second, our data reflect medication mentions and not antibiotics consumed, and the information if the parents or general practitioners (GP) discontinued antibiotics was limited. We did not analyze if those patients who did not receive antibiotics in PED were further prescribed any at the GP’s office as we wanted to clarify the situation in PED. Moreover, it is important to note that our study period coincided with the COVID-19 pandemic, which could potentially influence our findings. Given the broad spectrum of pathogenesis associated with COVID-19, which affects various organ systems beyond the respiratory system, individuals with a main diagnosis of COVID-19 disease were not included in this study. Consequently, certain data pertaining to supplementary laboratory investigations, instrumental tests, and rates of unnecessary antibiotic prescriptions were not captured, thus potentially resulting in a slightly skewed assessment of medical overuse within our study.

## 5. Conclusions

Our study revealed an overuse of medical tests even though the majority of URTIs were triaged as non-urgent (i.e., the fourth triage category) and viral infections. We observed that factors such as a longer duration of fever or physician suspicion of bacterial infection were associated with the performance of CBC, CRP, and even urinalysis tests in patients with URTI symptoms. Low numbers of non-urgent URTI cases saw even blood and urine cultures and visual testing, such as abdominal ultrasounds or chest X-rays, performed. Some of the clear viral cases were prescribed antibiotics unnecessarily. The main factors that influenced antibiotic prescription rates among all the patients were a younger age (1–4 years), suspected bacterial infection like tonsillitis, CRP values higher than 30 mg/L, as well as CBC or urinalysis test performance. The majority of non-urgent URTI cases were discharged from PED, and the third or second triage category were the main factors which determined 24 h observation rates in PED. Interventions are needed to improve the appropriateness of the use of emergency services in Lithuania.

## Figures and Tables

**Table 1:** General characteristics of the study population.

Parameters	Total (*n* = 410)	
Gender (female) (*n* (%)):	200 (48.8)	
Median age (year (IQR)):	2 (1–4)	
Age groups (*n* (%)):	<6 mo.	26 (6.3)
	6–11 mo.	51 (12.4)
	1–4 y.o.	252 (61.5)
	5–11 y.o.	61 (14.9)
	12–18 y.o.	20 (4.9)
Triage categories (*n* (%)):	2nd	3 (0.7)
	3rd	50 (12.2)
	4th	356 (86.8)
Time of arrival from the onset of fever (hours) (*n* (%)):	<24	55 (13.4)
	24–48	127 (31.0)
	≥48	182 (44.4)
Vital signs in PED:	RR (brpm (IQR))	26 (24–28)
	Tachypnoea (*n* (%))	1 (0.2)
	Mean fever +/− SD	37.3 +/− 0.9
	HR (bpm (IQR))	128 (119.8–140.0)
	Tachycardia (*n* (%))	20 (4.9)
	SpO_2_ (% (IQR))	98 (96–98)
	SpO_2_ < 94%	7 (1.7)
Auscultation findings (*n* (%)):	Normal	362 (88.3)
	Pathology (rales, crackles)	48 (11.7)
Final diagnosis (*n* (%)):	Nasopharyngitis, pharyngitis	309 (75.4)
	Tonsillitis	71 (17.3)
	Laryngitis	30 (7.3)
Origin of the infection (*n* (%)):	Bacterial	40 (9.8)
	Viral	308 (75.1)
	Not-specified	62 (15.1)
Antibiotic treatment (*n* (%)):	86 (20.1)	
Outcomes (*n* (%)):	Discharged	382 (93.2)
	Short stay	23 (5.6)
	Hospitalization	5 (1.2)

Abbreviations—*n*, number; IQR, interquartile range; SD, standard deviation; PED, pediatric emergency department; mo., months; y.o., years old; RR, respiratory rate; brpm, breaths per minute; HR, heart rate; bpm, beats per minute; and SpO_2_, oxygen saturation.

**Table 2:** Results of the diagnostic laboratory and instrumental tests performed in PED.

Diagnostic Test	Total (*n* = 410)		
CBC (*n* (%)):	307 (74.9)		
CBC result:	Leukocytes	Median (×10^9^/L (IQR))	9.8 (7.1–13.7)
		Normopenia (*n* (%))	217 (70.7)
		Leukocytosis (*n* (%))	46 (11.2)
	Neutrophils	Median (×10^9^/L (IQR))	5.2 (3.1–8.8)
		Normopenia (*n* (%))	205 (50)
		Neutrophilia (*n* (%))	92 (22.4)
CRP (*n* (%)):	312 (76.1)		
CRP result:	CRP (mg/L (IQR))	13.8 (5.0–36.8)	
	<60 mg/L (*n* (%))	275 (67.1)	
	>60 mg/L (*n* (%))	37 (9.0)	
Urinalysis (*n* (%)):	110 (26.8)		
Urinalysis result (*n* (%)):	Normal	100 (90.9)	
	Pathology (contamination)	10 (9.1)	
Urine culture (*n* (%)):	3 (0.7)		
Urine culture result (*n* (%)):	Normal	1 (33.3)	
	Pathology (contamination)	2 (66.7)	
Blood culture (*n* (%)):	1 (0.2)		
Blood culture result (*n* (%)):	Normal	1 (100.0)	
Rapid strep test (*n* (%)):	17 (4.1)		
Rapid strep test result (*n* (%)):	Normal	2 (11.8)	
	Pathology	15 (88.2)	
Chest X-ray (*n* (%)):	30 (7.3)		
Chest X-ray result (*n* (%)):	Normal	25 (83.3)	
	Suspected infiltration	2 (6.7)	
	Peribronchial changes	3 (10.0)	
Abdominal or/and chest US (*n* (%)):	19 (4.6)		
Abdominal or/and chest US result (*n* (%)):	Normal	19 (100)	

Abbreviations—*n*, number; US, ultrasound; CBC, complete blood count; CRP, C-reactive protein; and IQR, interquartile range.

**Table 3:** Associations between the age, gender, triage, origin of the infection, fever duration, and most common laboratory tests.

		O.R.	95% C.I.		*p*-Value
			Lower	Upper	
**CBC**	Age	1.911	0.351	10.409	0.454
	Gender	0.648	0.355	1.183	0.158
	Triage	1.533	0.594	3.961	0.377
	Origin of the infection	**9.314**	1.244	69.715	**0.030**
	Fever duration	**2.564**	1.182	5.563	**0.017**

**CRP**	Age	1.497	0.272	8.227	0.643
	Gender	**0.420**	0.219	0.805	**0.009**
	Triage	1.030	0.413	2.571	0.950
	Origin of the infection	**8.567**	1.140	64.357	**0.037**
	Fever duration	**2.900**	1.289	6.525	**0.010**

**Urinalysis**	Age	1.158	0.234	5.722	0.857
	Gender	1.055	0.616	1.808	0.845
	Triage	0.000			0.999
	Origin of the infection	**0.446**	0.211	0.942	**0.034**
	Fever duration	1.161	0.678	1.988	0.586

Abbreviations—CBC, complete blood count; CRP, C-reactive protein; and 95% C.I., 95% confidence interval. Significant values are presented in bold.

**Table 4:** Laboratory and instrumental tests according to the triage category, origin of the infection, and final diagnosis.

**Diagnostic Test**	**Total (*n* = 410)**	**Triage Category**			***p*-Value**
		**2 (*n* = 3)**	**3 (*n* = 50)**	**4 (*n* = 356)**	
**CBC (*n* (%)):**	307 (74.9)	3 (100)	43 (86.0)	260 (73.0)	0.085
**CRP (*n* (%)):**	312 (76.1)	3 (100)	42 (84.0)	266 (74.7)	0.220
**Urinalysis (*n* (%)):**	110 (26.8)		18 (36.0)	91 (25.6)	0.170
**Urine culture (*n* (%)):**	3 (0.7)			3 (0.8)	0.799
**Blood culture (*n* (%)):**	1 (0.2)		1 (2.0)		**0.027**
**Rapid strep test (*n* (%)):**	17 (4.1)		3 (6.0)	14 (3.9)	0.740
**Chest X-ray (*n* (%)):**	30 (7.3)		3 (6.0)	27 (7.6)	0.818
**Abdominal or/and chest US (*n* (%)):**	19 (4.6)		3 (6.0)	16 (4.5)	0.830
					
		**Origin of the infection**			
		**Bacterial (*n* = 40)**		**Non-bacterial (*n* = 370)**	
**CBC (*n* (%)):**	307 (74.9)	39 (97.5)		268 (72.4)	**0.001**
**CRP (*n* (%)):**	312 (76.1)	39 (97.5)		273 (73.8)	**0.001**
**Urinalysis (*n* (%)):**	110 (26.8)	18 (45.0)		92 (24.9)	**0.006**
**Urine culture (*n* (%)):**	3 (0.7)	2 (5.0)		1 (0.3)	**0.001**
**Blood culture (*n* (%)):**	1 (0.2)			1 (0.3)	0.742
**Rapid strep test (*n* (%)):**	17 (4.1)	6 (15.0)		11 (3.0)	**0.001**
**Chest X-ray (*n* (%)):**	30 (7.3)	6 (19.6)		24 (6.5)	0.050
**Abdominal or/and chest US (*n* (%)):**	19 (4.6)	2 (5.0)		17 (4.6)	0.908
		**Final diagnosis**			
		**URTI (*n* = 309)**	**Tonsillitis (*n* = 71)**	**Laryngitis (*n* = 30)**	
**CBC (*n* (%)):**	307 (74.9)	233 (75.4)	66 (93.0)	8 (26.7)	**0.001**
**CRP (*n* (%)):**	312 (76.1)	238 (77.0)	66 (93.0)	8 (26.7)	**0.001**
**Urinalysis (*n* (%)):**	110 (26.8)	82 (26.5)	25 (35.2)	3 (10.0)	**0.032**
**Urine culture (*n* (%)):**	3 (0.7)	2 (0.6)	1 (1.4)		0.705
**Blood culture (*n* (%)):**	1 (0.2)	1 (0.3)			0.849
**Rapid strep test (*n* (%)):**	17 (4.1)	3 (1.0)	14 (19.7)		**0.001**
**Chest X-ray (*n* (%)):**	30 (7.3)	26 (8.4)	4 (5.6)		0.201
**Abdominal or/and chest US (*n* (%)):**	19 (4.6)	18 (5.8)	1 (1.4)		0.127

Abbreviations—*n*, number; URTI, acute upper respiratory tract viral infection (including nasopharyngitis and pharyngitis); US, ultrasound; CBC, complete blood count; and CRP, C-reactive protein. Significant values are presented in bold.

**Table 5:** Associations between gender, age groups, fever duration, diagnosis, triage category, laboratory tests, and antibiotic prescription.

		Antibiotics Prescribed		*p*-Value
		Yes (*n* = 56)	No (*n* = 292)	
**Gender**	Male	28 (50.0%)	155 (53.1%)	0.672
	Female	28 (50.0%)	137 (46.9%)	
**Age group**	<6 months old	0 (0.0%)	21 (7.2%)	**0.018**
	6–11 months old	2 (3.6%)	46 (15.8%)	
	1–4 years old	41 (73.2%)	168 (57.5%)	
	5–11 years old	11 (19.6%)	43 (14.7%)	
	12–18 years old	2 (3.6%)	14 (4.8%)	
**Fever duration**	<24 h	10 (18.2%)	38 (14.8%)	0.617
	24–48 h	20 (36.4%)	84 (32.7%)	
	>48 h	25 (45.5%)	135 (52.5%)	
**Triage category**	2nd	1(1.8%)	1 (1.8%)	0.279
	3rd	8 (14.3%)	30 (10.3%)	
	4th	47 (83.9%)	261 (89.4%)	
**Laboratory tests**	CBC	54 (96.4%)	220 (75.3%)	**0.001**
	CRP	54 (96.4%)	223 (76.4%)	**0.001**
	CRP > 30 mg/L	44 (81.5%)	25 (11.2%)	**0.001**
	CRP > 60 mg/L	27 (50.0%)	1 (0.4%)	**0.001**
	Urinalysis	24 (42.9%)	69 (23.6%)	**0.003**
**Diagnosis**	URTI	13 (23.2%)	262 (89.7%)	**0.001**
	Tonsillitis	42 (75.0%)	4 (1.4%)	
	Laryngitis	1 (1.8%)	26 (8.9%)	

Abbreviations—*n*, number; CBC, complete blood count; CRP, C-reactive protein; and URTI—acute upper respiratory tract infection (including nasopharyngitis, pharyngitis. Significant values are represented in bold.

## Data Availability

The data presented in this study are available on request from the corresponding authors. The data are not publicly available due to patient privacy and confidentiality policy.

## Appendix A

**Table A1:** Analysis of the different factors associated with the discharge and 24 h observations.

		O.R.	95% C.I.		*p*-Value
			Lower	Upper	
**Discharged**	Age	0.521	0.022	12.236	0.686
	Gender	0.920	0.329	2.571	0.874
	Triage	**4.899**	1.650	14.540	**0.004**
	Duration of fever	2.425	0.769	7.644	0.131
	Auscultation	0.130	0.011	1.575	0.109
	Diagnosis	**14.007**	2.032	96.547	**0.007**
	CBC	0.422	0.005	37.348	0.706
	CRP	0.743	0.008	69.572	0.898
	Urine test	1.149	0.391	3.375	0.801
**24 h observation**	Age	1.094	0.608	1.970	0.764
	Triage	**3.043**	1.170	7.916	**0.023**
	Gender	1.035	0.439	2.438	0.937
	Duration of fever	0.181	0.020	1.613	0.126
	Auscultation	1.446	0.378	5.532	0.590
	Diagnosis	0.032	0.000	2.187	0.110
	CBC	0.608	0.026	14.038	0.756
	CRP	1.723	0.071	41.533	0.738
	Urine test	1.345	0.554	3.269	0.513

Abbreviations—CBC, complete blood count; CRP, C-reactive protein; O.R., odds ratio; and 95% C.I., 95% confidence interval. Significant values are represented in bold.
